# System-Size Effects on the Molecular Dynamics Simulation of an All-Aromatic Liquid Crystal

**DOI:** 10.1021/acsphyschemau.6c00022

**Published:** 2026-05-18

**Authors:** Henry Adenusi, Francesco Vita, Luca Muccioli, Matteo Lanciotti, Oriano Francescangeli

**Affiliations:** † Department of Science and Engineering of Materials, Environment and Urban Planning, Marche Polytechnic University, Via Brecce Bianche, 60131 Ancona, Italy; ‡ Department of Industrial Chemistry, University of Bologna, Via Gobetti 85, 40129 Bologna, Italy

**Keywords:** liquid crystal, molecular dynamics, system size, diffusion coefficients, order parameter

## Abstract

Atomistic molecular dynamics simulations are presented for a prototypical all-aromatic calamitic liquid crystal that has recently emerged as a benchmark system for testing theories of nematic order. By performing simulations with progressively larger systems, up to an unprecedented total of 12,600 molecules, we investigate the impact of system size on the structural and dynamic properties of the material in the nematic and smectic A mesophases. Our results demonstrate that using a number of molecules on the order of 10^4^ not only improves statistical sampling, thereby reducing uncertainty in all the computed quantities, but is also necessary to minimize finite-size and boundary-condition effects. This, in turn, enables unbiased estimates of key thermodynamic properties (such as transition temperatures and enthalpies, order parameters, and diffusion coefficients) and allows for a correct determination of the nature of the phase transitions (first- versus second-order). Moreover, we emphasize that large simulation boxes are essential to capture mesoscale structural features with characteristic length scales of several tens of nanometres, underscoring the unique capability of atomistic molecular dynamics simulations to complement experimental studies.

## Graphical abstract

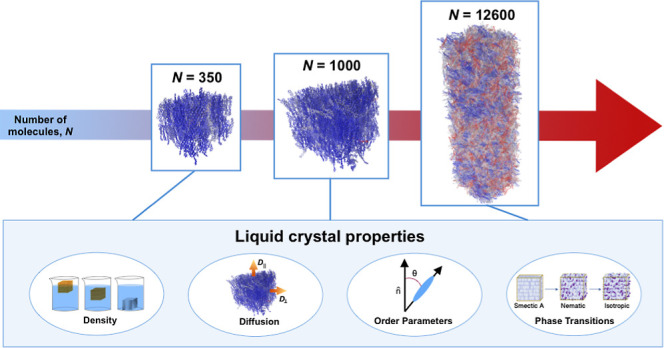

## 1 Introduction

Computational modeling has emerged as a viable technique for the description of complex condensed phases of liquid crystals (LCs), providing insights into their spontaneous molecular organization and dynamics.^1^ The defining features of LC phases depend critically on their mesoscale structure, whose characteristic size is smaller than that of a typical sample but still much larger than the molecular dimension. Furthermore, many LC phases exhibit a hierarchy of order parameters over length scales spanning several orders of magnitude. Consequently, realistic computational modeling of LC systems must necessarily include a large number of molecules. This requires a balance between the size of the simulated system, the computational requirements (CPU time and hardware resources), and the accuracy of the model. In this regard, coarse-grained (CG) approaches represent mesogen molecules as made of one or few rigid bodies, therefore significantly reducing the computational complexity.^2–4^ They can be used to highlight the emergence of specific phase properties, but generally cannot provide a reliable quantitative prediction of the whole phase behavior.^5^ By contrast, atomistic simulations can account for the crucial role of molecular conformations and flexibility in determining the mesophase behavior and macroscopic properties^6–8^ and have been used to characterize the temperature dependence of order–disorder transitions across the smectic (Sm), nematic (N) and isotropic (I) phases.^3,9–18^ Notably, molecular dynamics (MD) simulations have been employed to accurately elucidate a range of structural and dynamical features in LCs including order parameters and phase transition temperatures, density, diffusion coefficients, nuclear magnetic resonance (NMR) dipolar couplings,^19,20^ with interpretation complementary to experiments.^21^

Along with the level of detail of the potential energy function, also the selected cell volume *V*, the associated number of molecules *N*, and the spanned time scale can impact the ability of MD simulations to capture and predict the LC properties.^10,22,23^ Smaller samples are less computationally intensive, thus allowing for faster simulations and the exploration of a wide range of parameters, though they are subject to larger fluctuations and are more sensitive to boundary- and finite-size effects. While larger fluctuations result in a larger statistical uncertainty affecting the computed values, with the corresponding standard deviations (SDs) varying as *N* ^–1/2^, boundary- and finite-size effects results in a systematic dependence of the computed average values on *N*. This reflects the distance of the simulated system from the macroscopic system and the influence of boundary constraints, leading to results that may not reproduce the actual macroscopic behavior.^24–26^ Small systems can induce artificial ordering due to limited space for the molecules to orient/move, leading to spurious long-range correlations and difficulties in locating phase transition temperatures. Regarding boundary effects,^21^ though periodic boundary conditions (PBCs) can be considered a much milder form of confinement with respect to an impenetrable wall, they nonetheless affect the simulation results in small systems.^27–29^ On the other hand, larger systems provide a more precise and accurate representation of the LC bulk behavior, but necessitate longer simulation times to achieve equilibration of long-range properties (sufficient mixing and reaching a stable monodomain state) and require additional computational resources. Moreover, some authors have pointed out that, in locating a weakly first-order transition, increasing the system size better reproduces the free energy barrier between the two bulk states (e.g., nematic and isotropic), leading to a more realistic representation of the transition but also requiring longer simulation times for its assessment due to persistent metastable states and hysteresis.^30,31^

In the past, the effect of the system size on the simulation of LC systems has been investigated in the framework of CG models.^6,8,26,33^ Here, we explore the effects of the system size in MD modeling of LCs by comparing the results of simulations performed with an increasing number of molecules, up to an unprecedented maximum exceeding *N* = 10^4^ (corresponding to more than 7 × 10^5^ atoms). The investigated system is the 2,6-biphenyl naphthalene (PPNPP, Figure 1), a member of the unconventional class of all-aromatic calamitic LCs that have recently attracted great interest as archetypal rigid, rod-like nematogens.^32,34–37^ As such, they can be examined to scrutinize the fundamental theories that describe nematic order.^35^ In addition, their extended conjugated structure makes them relevant as prototypical derivatives for organic electronic technologies.^32,38,39^ The use of simulations on these systems is further justified by the challenges of experimental characterizations, due to their very high temperature *N* range (>400 °C), tendency to sublimate, and risk of sample decomposition.

**Figure 1:**
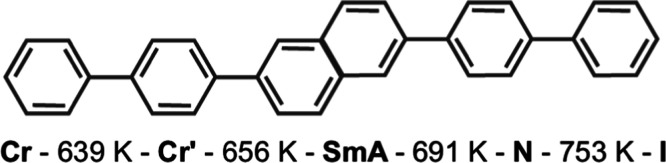
Molecular structure of PPNPP and phase sequence as obtained from differential scanning calorimetry at a heating rate of 20 °C min^–1^.^32^

Recent experimental and computational investigations for PPNPP suggest an unexpected propensity for local molecular layering within the N phase (cybotaxis, i.e. a SmA-like stratification within nanosized clusters of mesogens),^40,41^ which rises as the temperature declines.^32,37^ This unconventional nanostructure could explain the observed deviations of the orientational and positional order parameters from theoretical predictons.^37^

Besides providing structural information, MD simulations represent a valuable tool to investigate dynamical features. In several technological applications, interest lies in understanding the mesogen mobility as it can affect important properties such as ion transport.^42–44^ The simulated translational diffusion tensor components can be compared with those determined by a variety of experimental methods, e.g. electron spin resonance, NMR and quasi elastic neutron scattering (QENS).^11,22,45–48^ Yeh and Hummer reported that the simulated self-diffusion coefficients depend significantly on the system size with an underestimation of ∼10% when analyzing a periodically replicated cubic simulation cell.^48^ As a result, meaningful comparisons between simulated and experimental transport properties require correcting for these systematic errors and using suitable cell sizes.^22^

Toward progressing the understanding of system size effects on the structural and dynamical properties of simulated LCs, here we compare a series of three simulations performed with *N* = 350, 1000, and 12,600, with adequate time-scales to sample dynamical processes across the entire mesophase range, from the Sm to the I phase (Figure 2). By increasing the value of *N*, larger scale MD simulations provide the benefits of characterizing the spontaneous structural organization on a bulk scale, reducing finite size effects and improving statistical sampling. The temperature dependence of the phases is examined focusing on the density, phase transitions, orientational and positional order parameters. Lastly, the particle dynamics is analyzed computing the anisotropic translational diffusion coefficients that characterize the fluidity of this class of LCs.

**Figure 2:**
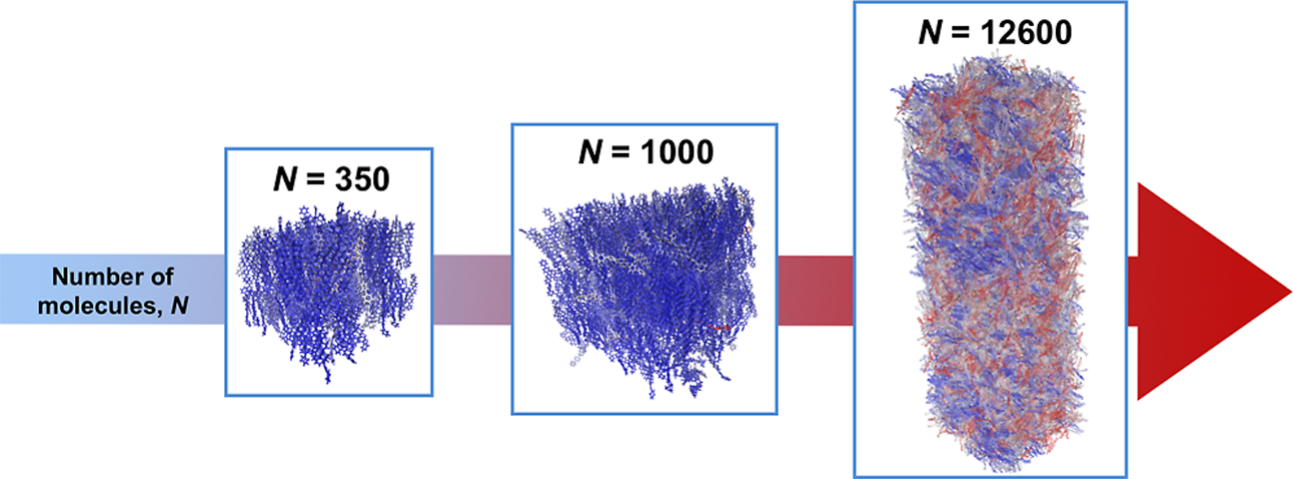
Representative MD simulations snapshots of PPNPP with increasing number of molecules and temperature: from left to right, *N* = 350 at 670 K (smectic A), *N* = 1000 at 710 K (nematic) and *N* = 12,600 at 750 K (isotropic). The molecules are color coded in relation to the orientation of their reference axis **u** with respect to the phase director **n**, ranging from blue (parallel) to red (perpendicular).

## 2 Methods

Atomistic MD simulations were performed with the NAMD software^49^ at constant atmospheric pressure and temperature using the standard time step of 1 fs, Berendsen barostat and thermostat.^49,50^ The particle Mesh Ewald (PME) method^49^ was used to compute the Coulomb interactions with PBCs, with a grid spacing of 1.5 Å and a cutoff of 12 Å for the calculation in the direct space, as well as for truncating Lennard–Jones interactions. The AMBER/GAFF parameters were employed, with atomic charges required for the force field obtained with density functional theory (DFT) calculations using Gaussian16 at the B3LYP/6-311++G­(d,p) level of theory, with the electrostatic potential (ESP) method.^51,52^ The NAMD topology and input files are reported in Sections S1 and S2 of the Supporting Information. Note that the force field was validated in a prior work^37^ and shown to adequately reproduce the experimental N–I and SmA–N transition temperatures for a sample of *N* = 350 molecules.

The NAMD software utilizes a parallel structure whereby three main types of calculations are performed, namely bonded interactions, short-range nonbonded interactions, and long-range electrostatic interactions handled via the PME algorithm. While the complexity of a pairwise computation of all nonbonded interactions would scale as *O*(*N* ^2^), the PME method reduces the computational cost to *O*(*N* log *N*) by explicitly calculating pairwise nonbonded interactions within a specified cutoff radius and accounting for longer-range contributions through reciprocal-space Ewald interpolation. After a few preliminary tests performed on the Cineca high performance computing (HPC) facility, all the simulations were performed on our in-house server featuring two Intel­(R) Xeon­(R) Gold 6418H CPUs and 1024 TB of RAM. In terms of the computational cost for each simulation, we report the following benchmark times obtained with 48 CPU cores at 700 K: 0.010 days/ns (100 ns/day) for *N* = 350, 0.017 days/ns (59 ns/day) for *N* = 1000, 0.14 days/ns (7 ns/day) for *N* = 12,600.

### 2.1 *N* = 350

Preliminary information on the LC mesophases of PPNPP was reported in a previous paper.^37^ It was obtained using a small system of *N* = 350 molecules contained in a cubic box with PBCs and box sides of ∼60 Å. MD simulations were equilibrated for 40 ns and the production runs ranged from 35 to 40 ns. To capture the main features of the PPNPP phase map, we carried out a complete cooling down sequence from the disordered I phase at 800 K to the crystalline phase at 650 K. Those results represented the basis for the study of larger systems, as detailed below.

### 2.2 *N* = 1000

This system was prepared with *N* = 1000 molecules in a cubic box and equilibrating the sample for 10 ns. Subsequently, MD simulation production runs were performed for 45–50 ns. Based on the results obtained for *N* = 350, the sample was heated from 670 K (in the Sm phase) to 780 K (in the I phase).

### 2.3 *N* = 12,600

The largest sample was prepared by replicating the smaller sample (*N* = 350) at 690 K, choosing as initial configuration one with the cell *y*-axis aligned parallel to the nematic director, to produce a bulk system of *N* = 12,600 molecules, extending in the *x*, *y*, *z* dimension by 2 × 9 × 2. We used a longer extension along the *y*-axis as this permits having approximately the same number of molecules in each direction. Equilibration runs were performed for 10 ns and production runs of 15–25 ns, starting from the sample equilibrated at 690 K and either cooling down to 670 K or heating up to 780 K.

For any *N*, we verified that the chosen equilibration times were sufficient to equilibrate the system (see Section S3 of the Supporting Information for further details). We also observed that the initial periodicity of the largest system was quickly lost. The most likely explanation for the relatively low equilibration times is that PPNPP LC phases occur at very high temperatures where the translational self-diffusion coefficients are remarkably large (∼10^–9^ m^2^/s).

### 2.4 Analysis

Statistical structural descriptors including the orientational and positional order parameters, phase transitions temperatures, enthalpies and translational diffusion coefficients were calculated with established computational algorithms^1^ using in-house developed analysis codes. The liquid crystalline phase director **n**(*t*) was calculated for each frame by diagonalizing the order matrix,^1^ with the molecular orientation defined by the long molecular axes **u**(*t*), i.e. the axis with the smaller moment of inertia. Then, the orientational order parameters $\left\langle P_{2}\right\rangle =\left\langle \frac{3\cos^{2}\theta -1}{2}\right\rangle$ and $\left\langle P_{4}\right\rangle =\left\langle \frac{35\cos^{4}\theta -{30\cos}^{2}\theta +3}{8}\right\rangle$ were obtained by averaging over time and molecules *i* the values of cosθ_i_ = **n**(*t*)·**u**_*i*_(*t*). The positional order parameter τ_1_ = ⟨cos­(2π*z*/*d*)⟩ was obtained from the molecular centers of mass following the procedure detailed in ref 11 The isotropic diffusion coefficient *D*_iso_ and the corresponding values along the phase director and perpendicular to it, *D*_∥_ and *D*_⊥_, were calculated from the mean square positional displacements, following the procedure detailed in Section S4 of the Supporting Information.

For all observables, standard errors of the means (SEs) were computed through the block averaging method.^53,54^ In practice, the time series is divided into *N*_b_ blocks of equal length; for each block, the observable of interest is averaged, producing a set of block means and standard deviations that are statistically independent, provided that the blocks are long enough. Hence, block length is gradually increased until the SE, estimated as $\sigma_{b}/\sqrt{N_{b}}$ with σ_b_ being the SD of the block averages, converges to the correct statistical uncertainty. For the diffusion coefficients, the application of this method is particularly cumbersome and would ideally require longer trajectories. We applied it to all systems at *T* = 730 K, as described in Section S4 of the Supporting Information, and evaluated the uncertainty on *D*_ii_ being of the order 1%. As a consequence, we decided to assign an indicative uncertainty of 2% to all our diffusion coefficient data.

Although we performed simulations at constant temperature, small fluctuations are inherently present in the system. For the smaller system (*N* = 350) at all temperatures, we evaluated a SD of ∼3 K and a SE on the temperature value of ∼0.02 K. We considered this uncertainty negligible.

## 3 Results and Discussion

### 3.1 Density and Phase Transitions

Figure 3 reports the simulated density as a function of temperature *T* for the three investigated samples. In agreement with the experimental phase map (Figure 1), the simulated systems exhibit three different phases across the investigated temperature range as evidenced by the changes of the slope of the curves: on heating these are the SmA, N and an I phases, as confirmed by the analysis of the relevant order parameters discussed in the following section. The system size has little impact on the density of the system in the N phase: the simulated densities are very similar, with a very slight tendency for the smaller systems to display higher values. This trend becomes stronger in the high temperature I phase and low temperature Sm phase. From the temperature dependence of the density, the SmA-N and N–I phase transition temperatures can be roughly located for all sample sizes in the range *T*_SmA‑N_ = (695 ± 5) K and *T*_N–I_ = (750 ± 10) K, respectively, with an apparent tendency of both to move to the lower side of the range on increasing *N*. A more precise determination of the transition temperatures was obtained from the temperature dependence of the corresponding order parameters as detailed in Section S5 of the Supporting Information: the results, summarized in Table 1, evidence for both phase transitions a downshift of the order of 10 K on passing from *N* = 350 to *N* = 12,600, much larger than the reported uncertainties. Overall, the results are in good agreement with the experimental values obtained from combined differential scanning calorimetry (DSC) and polarization optical microscopy (POM).^37^ The minor discrepancies can be attributed to the combined effect of the experimental uncertainty in this class of compounds (caused by many factors, such as the heating/cooling rate, the thermal history of the sample and related hysteresis effects, sample degradation, etc.)^32^ and, concerning MD simulations, to the inaccuracy of the force field.

**Figure 3:**
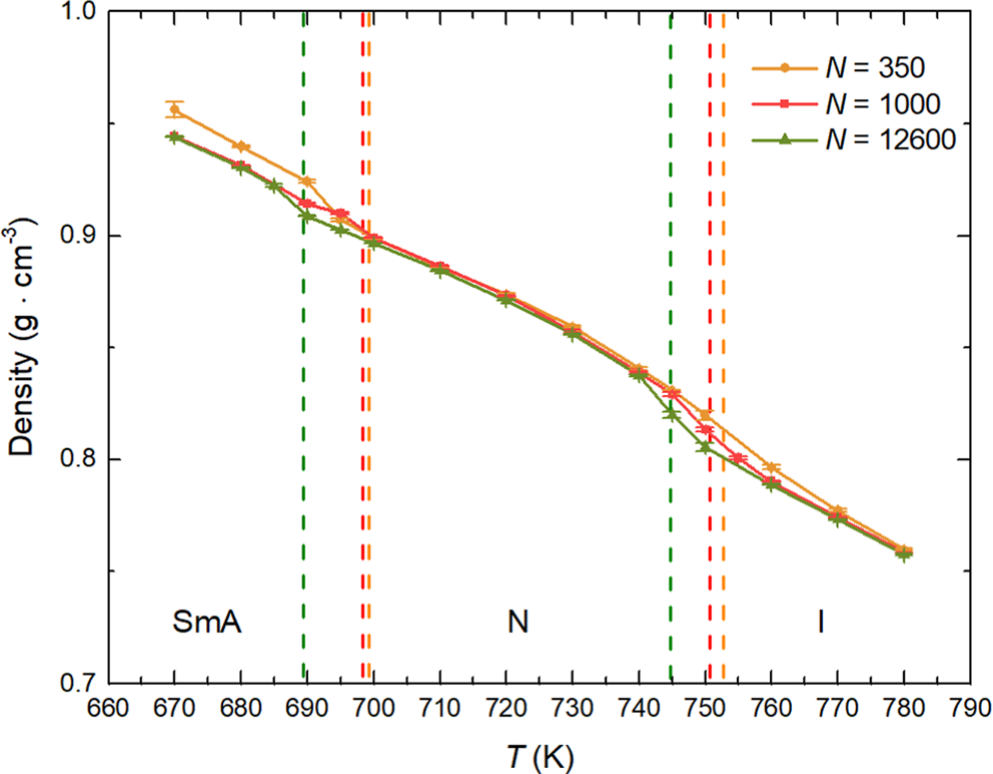
Simulated density as a function of temperature for each system: *N* = 350, *N* = 1000 and *N* = 12,600. Error bars indicate SEs (where not visible, they are smaller than data symbols). Vertical dashed lines indicate the transition temperatures as obtained from the analysis of the orientational and positional order parameters.

**Table 1:** Values of the Simulated and Experimental Phase Transitions Temperatures, Estimated from the Trends of the Order Parameters as Detailed in Section S5 of the Supporting Information, and Corresponding Transition Enthalpies^a^

	*N* = 350	*N* = 1000	*N* = 12,600	Exp
Transition temperature	*T* (K)	*T* (K)	*T* (K)	*T* (K)
N–I	752.8 ± 1.2	750.7 ± 0.6	744.7 ± 0.5	753 (heating)
				744 (cooling)
SmA-N	699.3 ± 1.4	698.3 ± 2.2	689.4 ± 0.4	691 (heating)
				670 (cooling)
Transition enthalpy	Δ*H* (kJ mol^–1^)	Δ*H* (kJ mol^–1^)	Δ*H* (kJ mol^–1^)	Δ*H* (kJ mol^–1^)
N–I	5.0 ± 1.3	4.5 ± 1.2	3.4 ± 0.4	1.6 (heating)
				2.6 (cooling)
SmA-N	1.5 ± 0.9	0.2 ± 0.8	0.0 ± 0.2	0

a Experimental values from ref 37.

Actually, the level of accuracy of the simulated transition temperatures with respect to experiment, reached here with the GAFF force field, is rather surprising, since many authors reported very large deviations from experimental values in similar studies with general force fields^12–14,16^ and the need of reparametrizing torsional degrees of freedom and intermolecular Lennard–Jones interactions to reduce them has been long since recognized in the field.^15,55,56^ Although we are unable to provide a clear-cut explanation for these fluctuating performances of general force fields, we note that most failures refer to rather flexible molecules with alkyl chains, while for all-aromatic compounds accuracy is typically acceptable.^36,37,57,58^

Another possible source of error is the difficulty in locating the exact transition point, especially for small system sizes. In fact, the transition is often smoothed by finite size effects, causing a nonvanishing relevant order parameter even in the higher temperature phase. Consequently, setting the transition point requires the choice of a criterium that inevitably entails some degree of arbitrariness. In our case, it was determined by analyzing the temperature dependence of the relevant order parameter (⟨*P*_2_⟩ and τ_1_ for the N–I and SmA-N phase transitions, respectively) as described in the following section and detailed in Section S5 of the Supporting Information. While the chosen methodology may slightly affect the absolute values of the transition temperatures, it has no effect on their dependence on the system size.

Table 1 also reports a comparison of the experimental transition enthalpies Δ*H* with those obtained from simulations. The system enthalpy was evaluated as *H*(*T*) = *U*(*T*) + *pV*(*T*), where *U*(*T*) and *V*(*T*) are the average values of the internal (kinetic and potential) energy and the box volume, respectively, and *p* is the external pressure of 1 atm. Figure 4 illustrates the behavior of enthalpy *H*(*T*) over the entire investigated temperature range. The transition enthalpy Δ*H* for both N–I and SmA-N transitions was calculated as the difference between the extrapolated linear trends above and below the corresponding transition. Details on the calculation of Δ*H* and the corresponding uncertainties are reported in Section S6 of the Supporting Information. For both phase transitions, the Δ*H* values decrease with the increasing system size, approaching the experimental data provided by DSC analysis. For the N–I phase transition, the relatively small values of Δ*H* point to its weakly first-order nature, in agreement with theoretical predictions. On the other hand, for the SmA-N transition, the much smaller values of Δ*H* exhibit a clear trend to zero on increasing *N*, with the value of Δ*H* becoming null within errors for the largest two systems, unequivocally reflecting the experimentally observed second-order nature of the SmA-N phase transition.

**Figure 4:**
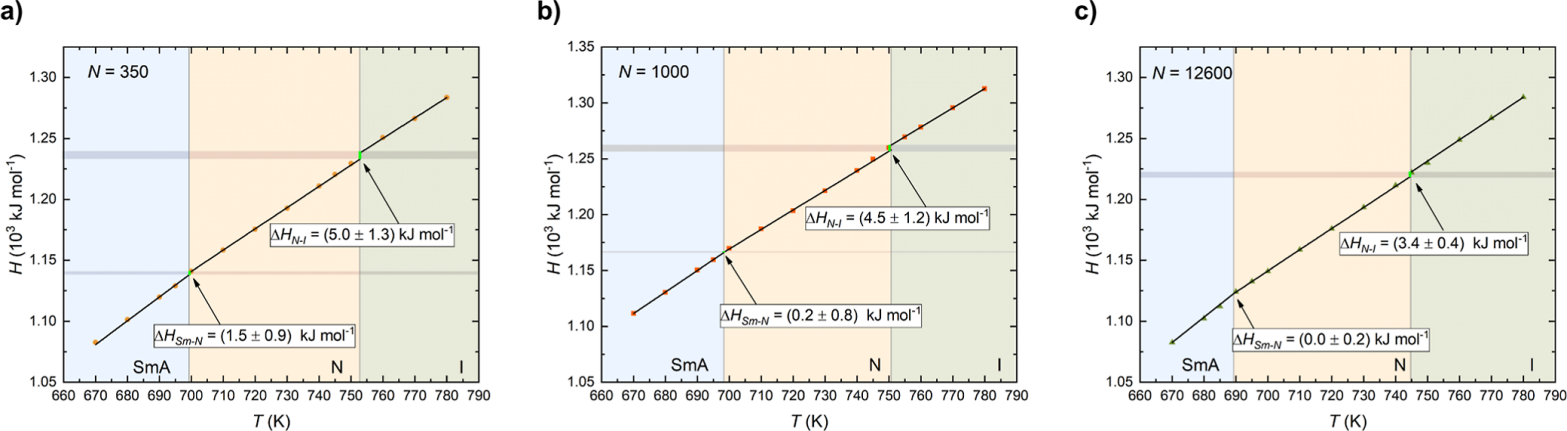
(a–c) Simulated molar enthalpy *H* as a function of temperature *T* and corresponding linear fit in each phase for different system sizes: (a) *N* = 350, (b) *N* = 1000, (c) *N* = 12,600. The shadowed horizontal bands highlight the extrapolated enthalpy gaps at the transition temperatures. SEs are smaller than data symbols.

### 3.2 Order Parameters

Figure 5 displays the temperature dependence of the orientational order parameters ⟨*P*_2_⟩ and ⟨*P*_4_⟩ over the entire explored temperature range for all the investigated sizes. In all cases we observe a small jump around *T* ∼ 690 K followed by a much more pronounced drop at *T* > ∼740 K, which correspond to the SmA-N and phase N–I transitions, respectively. For each sample size, the N–I transition temperature was identified with the center of a modified sigmoid function that best fits the temperature trend of ⟨*P*_2_⟩ in the transition range, as detailed in Section S5 the Supporting Information: this allowed us to assign the values of *T*_N–I_ reported in Table 1. The values obtained show a progressive decrease in the transition temperature as the system size increases.

**Figure 5:**
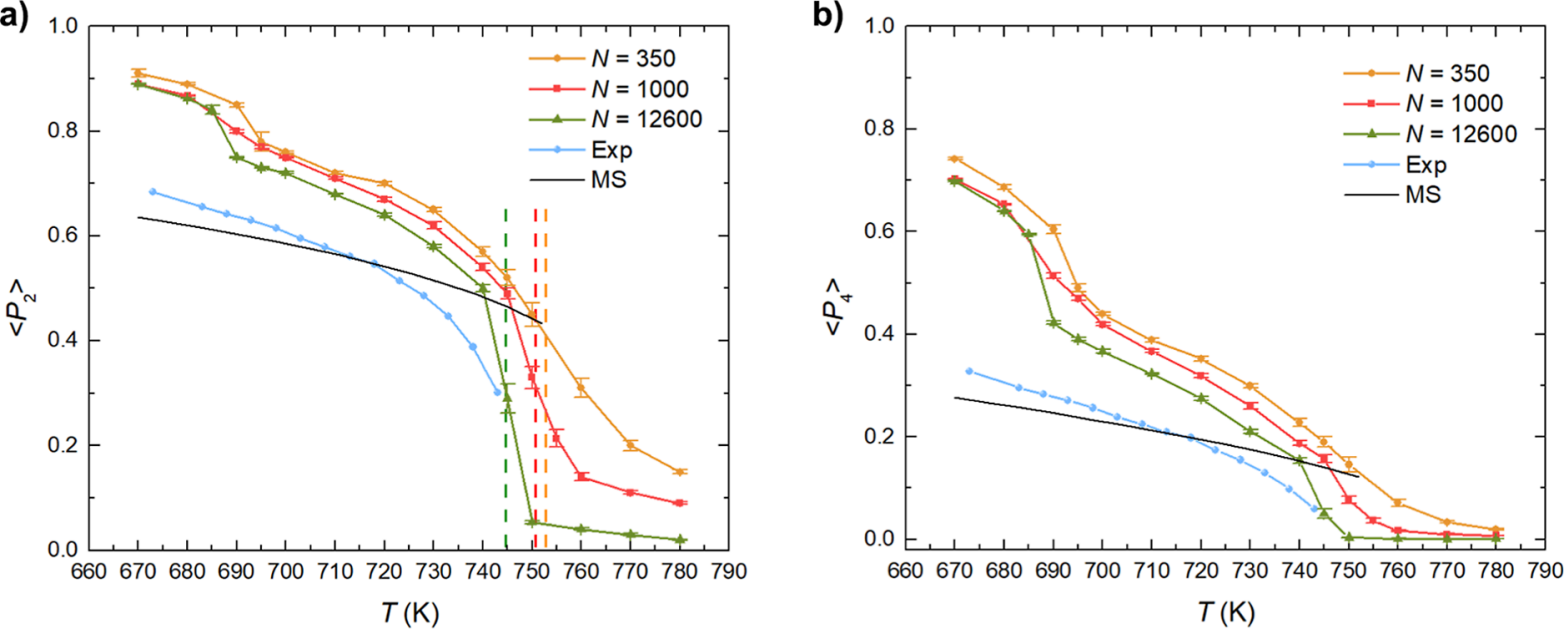
(a,b) Simulated orientational order parameters as a function of temperature *T* for *N* = 350, *N* = 1000 and *N* = 12,600 (yellow circles, red squares and green triangles, respectively), compared with those obtained from X-ray diffraction measurements (blue circles) and with the Maier–Saupe theoretical prediction (black line): (a) ⟨*P*_2_⟩ and (b) ⟨*P*_4_⟩. Error bars indicate SEs (where not visible, they are smaller than data symbols). In (a), the N–I transition temperature obtained from the analysis of each curve is indicated by a vertical dashed line of the same color.

A comparison of the plots in Figure 5 shows that, while the general trend below 740 K is similar for all system sizes differing only in the absolute values, which slightly decrease with increasing *N*, the three systems provide quite different descriptions of the N–I phase transition. For the smaller systems, ⟨*P*_2_⟩ is significantly different from zero even in the I phase (at 770 K: ⟨*P*_2_⟩ = 0.20 for *N* = 350, ⟨*P*_2_⟩ = 0.11 for *N* = 1000) resulting in a smooth transition, which however becomes markedly more abrupt as the system size increases (at 770 K: ⟨*P*_2_⟩ = 0.03 for *N* = 12,600), in agreement with the theoretical expectation for a first-order phase transition. This result highlights the need of a remarkably large number of molecules to accurately locate the N–I phase transition and determine its nature (first or second order),^59^ as already evidenced by previous MD simulation studies on 4-*n*-octyl-4 cyanobiphenyl and sexithiophene LCs, although with system sizes much smaller than our maximum.^11,57^ A similar trend is observed for ⟨*P*_4_⟩.

As a general trend, both nematic order parameters, ⟨*P*_2_⟩ and ⟨*P*_4_⟩, progressively decrease with increasing system size. However, even for *N* = 12,600, a significant difference persists between the simulated values and the experimental data obtained by X-ray diffraction (XRD) measurements^32^ (blue circles in Figure 5): whereas the general temperature dependence is well reproduced by the simulations, the experimental values are clearly lower than the simulated ones, with a gap that does not seem fillable by simply increasing the system size. This difference may be partially attributed to the nature of the XRD experimental data that also incorporate spatial inhomogeneities of the molecular director, therefore systematically providing underestimated values. In addition, different experimental techniques may provide different values of ⟨*P*_2_⟩, as observed for a similar all-aromatic LC.^12,36^ However, this discrepancy is also present when simulations are compared to the Maier–Saupe (MS) theoretical predictions (black continuous line in Figure 5), as also observed in similar simulated systems,^36,57^ suggesting that the choice of the force field may possibly play a role.

To gain deeper insight into the formation of the SmA phase when cooling down from the nematic, we also calculated the positional order parameter τ_1_ = ⟨cos­(2π*z*/*d*)⟩. It represents the first coefficient in the Fourier expansion of the probability *P*(*z*) of finding a molecule at a position *z* along the normal to the layers, with *d* ≈ 27.0 Å being the smectic layer spacing, essentially independent of the system size. The substantial coincidence of the simulated *d* value with the molecular length *L* ≈ 26.8–27.0 Å unambiguously confirms the orthogonal (SmA) nature of the smectic phase, in agreement with previous XRD data.^32^ To validate the robustness of our simulations, Figure 6 compares the values of τ_1_ for the different sample sizes. The overall temperature dependence remains approximately the same, irrespective of *N*: on heating from low temperatures, a steep decline in τ_1_ is observed for all the systems, indicative of the SmA-N phase transition. However, a clear tendency of the transition region to shift to lower values on increasing *N* is evident, in agreement with the trend of ⟨*P*_2_⟩ and ⟨*P*_4_⟩ previously discussed. Exact values for the SmA-N transition temperature were determined by fitting the temperature trends of τ_1_ with a modified sigmoid curve. In this case, in consideration of the second order character of the SmA-N phase transition, *T*_SmA‑N_ was identified with the point where the sigmoid decreases by 90% of its maximum value rather than with its center as done for the N–I transition (see Section S5 of the Supporting Information for additional details). Contrary to theoretical expectations, for all the investigated systems the values of τ_1_ remains significantly different from zero above the SmA-N transition, especially for the smaller systems. This evidence reflects the fact that small numbers of molecules tend to favor more ordered phases, resulting in order parameters that do not vanish above the corresponding phase transitions, as also observed for ⟨*P*_2_⟩ above *T*_N–I_. In the case of τ_1_, despite its residual value in the N phase appears strongly reduced as *N* increases, it persists with a value different from zero even for the largest system size. The persistence of this effect across the entire N range even for the largest sample suggests that it is a genuine physical effect, rather than an artifact arising from the box finite size. Accordingly, this finding corroborates the experimental evidence of the presence of short-range smectic order within the N phase (cybotaxis) suggested by XRD measurements.^32^

**Figure 6:**
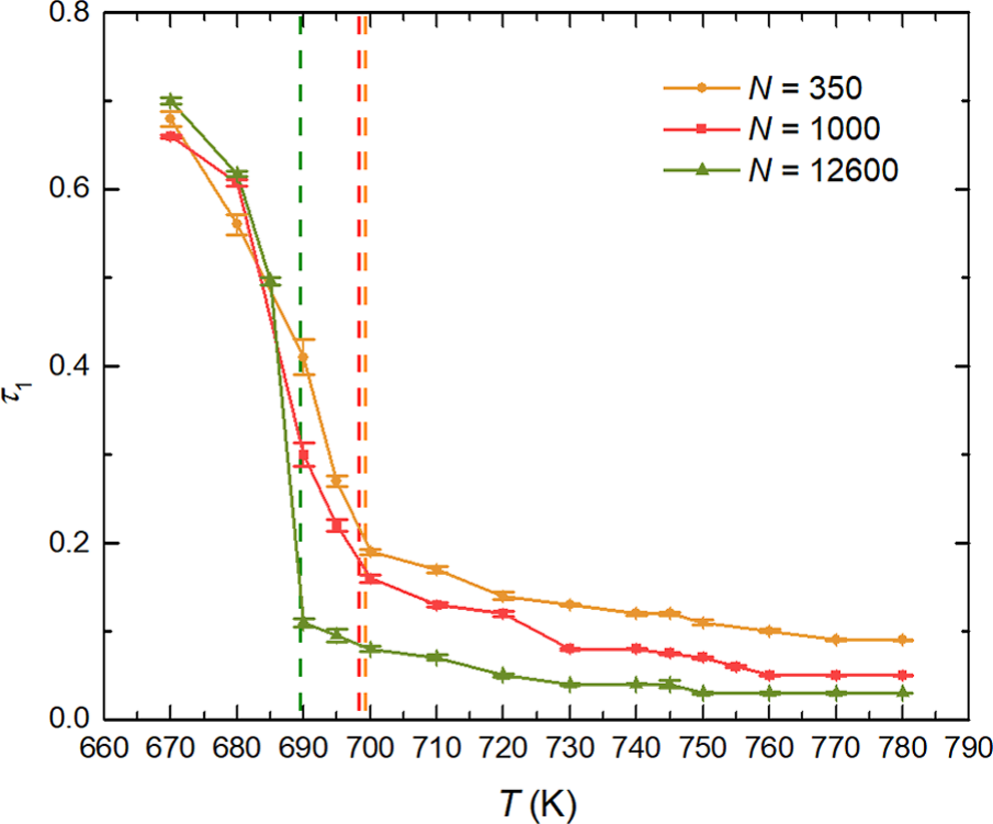
Positional order parameter τ_1_ as a function of temperature for *N* = 350, *N* = 1000 and *N* = 12,600. Error bars indicate SEs (where not visible, they are smaller than data symbols). The SmA-N transition temperature obtained from the analysis of each curve is indicated by a vertical dashed line of the same color.

### 3.3 Dynamical Properties

Besides influencing the structural properties, the system size also affects the dynamical behavior. To assess this effect, we compared the translational diffusion coefficients for the three system sizes. In the N phase, the translational diffusion coefficients are anisotropic with a uniaxial character: the molecules are on average aligned along the molecular director **n** and are expected to diffuse more easily along the director than orthogonally to it.^60^ The translational diffusion coefficients, shown in Figure 7, are on the order of 10^–9^ m^2^ s^–1^ with diffusion along the director proceeding faster with respect to the perpendicular direction in both the N and Sm phases, irrespective of the system size.

**Figure 7:**
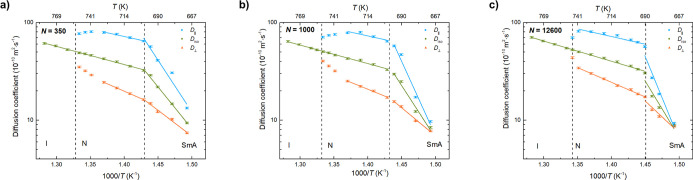
(a–c). Simulation results (points) and Arrhenius fits (continuous lines) in the I, N, SmA phases for the translational diffusion coefficients. The line extension covers the fitted range. Error bars indicate the estimated SEs.

The behavior of the isotropic diffusion, in both the I and the N phase, is known to follow an Arrhenius temperature dependence,^45^ i.e. $D_{iso}=D_{0}e^{-E_{a}/RT}$ , where *D*_0_ is the diffusion coefficient for temperature *T* → ∞, *E*_a_ is the molar activation energy required for molecules to overcome the potential barrier encountered while moving through the sample, and *R* is the gas constant. Several authors proposed theoretical models to quantify the dependence on the orientation order parameter of the diffusion tensor; for instance, in the model proposed by Chu and Moroi (CM),^61^ the anisotropic diffusion coefficients *D*_||_ and *D*_⊥_ in the N phase are expected to depend on the orientational order parameter ⟨*P*_2_⟩ as follows: $D_{∥}{=D}_{iso}\left[1+2\left\langle P_{2}\right\rangle \frac{1-\xi }{2\xi +1}\right]$ and $D_{⊥}=D_{iso}\left[1-\left\langle P_{2}\right\rangle \frac{1-\xi }{2\xi +1}\right]$ , with the parameter $\xi =\frac{\pi }{4Q}$ being inversely proportional to the aspect ratio *Q* = *L*/*d* ≈ 3.6, where *L* and *d* are the molecular length and diameter, respectively (the values of *Q* provided by simulations for each temperature are reported in Table S3). Accordingly, far from the N–I transition, where the temperature dependence of the orientational order parameter is relatively weak, *D*_||_ and *D*_⊥_ are also expected to follow the Arrhenius law. Table 2 summarizes the values of the activation energy *E*_a_ for the isotropic and anisotropic diffusion coefficients resulting from the Arrhenius fits of the simulated data (see Table S4 for the corresponding values of *D*_0_). For *D*_iso_ the fit includes data in both the I and the N phase, whereas for *D*_||_ and *D*_⊥_ it is limited to the N phase excluding the points too close to the N–I phase transition where the temperature dependence of the order parameter is stronger. Separate fits were performed across the SmA phase. The fits in the N phase show a good agreement with the simulated data (Figure 7), which becomes excellent for the largest sample size. For *N* = 12,600, the more accurate description of the N–I phase transition results in the diffusion coefficients following the Arrhenius law over a broader temperature range compared to the smaller samples. This is consistent with the N–I transition becoming sharper and narrower as the system size increases.

**Table 2:** Activation Energy *E*_a_ (kJ mol^–1^) and Corresponding Uncertainty as Obtained by Fitting *D*_iso_, *D*_|*|*_ and *D*_⊥_, for Each System Size, in the Isotropic-Nematic (N–I) and Smectic A (SmA) Regions

	*N* = 350			*N* = 1000			*N* = 12,600		
*E*_a_ (kJ mol^–1^)	*D*_iso_	D_||_	*D*_⊥_	*D*_iso_	D_||_	*D*_⊥_	*D*_iso_	D_||_	*D*_⊥_
**N–I**	35.7 ± 0.6	29.2 ± 2.9	58.10 ± 0.10	36.3 ± 0.9	29 ± 8	53.9 ± 2.3	39.5 ± 1.0	30 ± 4	57.6 ± 1.5
**SmA**	164 ± 7	203 ± 24	101 ± 7	205 ± 25	290 ± 40	110 ± 7	220 ± 50	320 ± 70	124 ± 27

In addition, with reference to the N phase, one can notice from Figure 7 that *D*_||_, and consequently *D*_iso_, exhibit a slight increase with increasing *N*. We verified that this effect does not depend on our choice to perform simulations at constant pressure rather than at constant volume, as discussed in Section S4 of the Supporting Information. Actually, our finding resembles that outlined by Yeh and Hummer^48^ for isotropic conventional liquids, where periodic boundary conditions are demonstrated to cause a reduction of the estimated translational diffusion inversely proportional to the length of one side of the simulation box (hence proportional to *N* ^–1/3^ in the case of a cubic box). For liquid-crystalline materials, this geometric effect is further exacerbated by the influence of *N* on the interplay between orientational and translational order.^62^ While an increase of the former favors diffusion along the director, a growth of the latter determines a potential barrier to the molecular motion across the layers. For large *N*, the residual value of the positional order parameter in the N phase becomes significantly smaller, as previously discussed, thus favoring translational motion along the director. Overall, these results underline the importance of performing simulations with a sufficiently large system size to reliably describe the dynamical properties.

Concerning the activation energies reported in Table 2, in the N phase, regardless of the sample size, we find *E*_a_^⊥^ > *E*_a_^iso^ > *E*_a_^||^, in agreement with the experimental results reported for some conventional nematics,^45,63^ which reflects the easier diffusional motion of the molecules parallel to the director rather than orthogonally.

On cooling down into the SmA phase, the diffusion anisotropy Δ*D* = *D*_||_ – *D*_⊥_ strongly reduces but remains positive. This result, consistent with those obtained from simulations on other all-aromatic mesogens,^36,57^ may appear somehow surprising as the Sm phase is generally regarded as a stack of two-dimensional liquid layers, thus favoring diffusion within the layers rather than across them. Nonetheless, our findings are in agreement with several experimental and simulation studies on systems where the SmA-N transition is weakly first or second order,^36,57,64^ suggesting that a sign-inversion of the diffusion anisotropy requires a more substantial energy barrier across the smectic layers, such as that encountered in tilted smectic phases.^63,64^ In the SmA phase, the model developed by Volino and coauthors^65^ predicts that *D*_⊥_ follows the same temperature dependence observed in the nematic, while *D*_||_ decreases more rapidly with decreasing temperature because of the onset of an energy barrier hampering the motion across the smectic layers. The fits of our simulated values across the SmA phase approximately conform with these theoretical predictions, especially for the largest system, with the activation energy *E*_a_^||^ showing an approximately 10-fold increase on entering the SmA phase and the consequent vanishing of the diffusion anisotropy Δ*D* at the lowest investigated temperature.

## 4 Conclusions

We performed a comprehensive computational characterization of a prototypical all-aromatic liquid crystal to investigate the system-size dependence of its structural and dynamical properties. Our results demonstrate that atomistic simulations with increasing *N*, reaching up to an unprecedented value of 12,600 molecules, captures spontaneous structural organization at the bulk scale, while simultaneously reducing finite-size and boundary-condition effects and improving statistical sampling. The density displays consistent trends across all of our samples, with a slight shift toward lower values for larger systems. Regarding the transition temperatures, the values obtained from the order parameter analysis exhibit a systematic dependence on *N*, with smaller systems exhibiting a clear tendency to favor more ordered phases. However, the determination of transition temperatures from the temperature dependence of the relevant order parameters is made more challenging for smaller systems because of the nonvanishing ⟨*P*_2_⟩ and *τ*_1_ values above the N–I and SmA–N transition temperature, respectively. In this perspective, increasing the size of the simulated ensemble turns out to be a key factor to accurately elucidate the exact transition position and, above all, its character (first vs second order). This is particularly evident for the N–I phase transition, whose actual first-order nature becomes manifest only for the largest simulated sample.

System-size effects are also reflected in the translational diffusion coefficients, with larger systems adhering more closely to the expected Arrhenius behavior across the I and N phases. However, the evidence of a “nematic-like” diffusion in the Sm phase, with *D*_||_ > *D*_⊥_ and *E*_a_^⊥^ > *E*_a_^iso^ > *E*_a_^||^ for all of the simulated samples, does not appear to be an effect of the limited system size. In fact, this diffusive behavior points to a weak potential barrier to molecular motion across the smectic layers, reflecting the second order nature of the SmA–N transition, and encourage further investigations, e.g. by NMR, for an experimental verification of the simulated diffusional properties of the PPNPP mesophases.

The findings reported in this paper underscore the importance of simulating sufficiently large systems to yield accurate predictions of experimental behavior. Fortunately, recent advances in computing technology have made it possible to perform atomistic MD simulation on systems of considerable size while still maintaining reasonable computational times. In addition to increasing the system dimension, ameliorating the simulation quality would require further extension of the simulation time scales. As this can greatly elevate the computational cost, a compromise between the computational requirements and the objective of the simulation is generally the ideal approach. Our results demonstrate that a system size on the order of *N* ∼ 10^4^ (with simulation times of a few tens of nanoseconds for our system of fast-moving molecules) provides a reasonable compromise between the above-mentioned competing demands. However, the choice of the optimal system size is obviously dictated by the available resources (time and computational power), the peculiarities of the simulated system, and the objectives of the research effort, in particular in terms of the desired accuracy close to the transition regions. In cases where very large MD simulations are less practical, e.g. due to the need of longer simulation times, an alternative approach is the use of less computationally intensive CG models, despite the inherent trade-off in accuracy.^26,33^

Besides improving the reliability of simulation results, a larger system size may allow the investigation of mesoscale structural details that cannot otherwise be captured as their characteristic length scale exceeds the dimension of smaller samples. For instance, evaluating the presence of short-range positional order (cybotaxis) within the N phase of PPNPP, anticipated by previous XRD studies^32^ and possibly confirmed by the nonvanishing value of *τ*_1_ in the N phase of our simulations, would require a system size significantly larger than the characteristic cybotactic cluster dimensions (at least ∼5 molecular lengths in the longitudinal direction and ∼5 average intermolecular distances in the transverse directions). Our simulations represent an important step in this direction, providing a valuable investigation tool to complement experimental studies on nanoscale structural order.

## Supplementary Material



## Data Availability

The data supporting this study’s findings are available from the corresponding authors upon reasonable request. Analyzed data supporting this article has been included as part of the ESI.

## Notes

Topology file in CHARMM format; example NAMD input configuration file; equilibration of density, orientational and positional order parameter at *T* = 690 K and at 750 K; methodology for the computation of translational diffusion coefficients; methodology for the determination of transition temperatures and enthalpies; values of simulated aspect ratio as a function of temperature; preexponential factors *D*_0_ from Arrhenius fits of diffusion coefficients (PDF)

## References

[1] Zannoni, C. Liquid Crystals and Their Computer Simulations; Cambridge University Press, 2022.

[2] Wilson M. R., Yu G. (2023). Computer Simulations of a Twist Bend Nematic (NTB): A Coarse-Grained
Simulation of the Phase Behaviour of the Liquid Crystal Dimer CB7CB. Crystals.

[3] Walker M., Wilson M. R. (2016). Simulation Insights
into the Role of Antiparallel Molecular
Association in the Formation of Smectic A Phases. Soft Matter.

[4] Francescangeli O., Stanic V., Torgova S. I., Strigazzi A., Scaramuzza N., Ferrero C., Dolbnya I. P., Weiss T. M., Berardi R., Muccioli L., Orlandi S., Zannoni C. (2009). Ferroelectric
Response and Induced Biaxiality in the Nematic Phase of a Bent-Core
Mesogen. Adv. Funct. Mater..

[5] Orlandi S., Muccioli L., Berardi R. (2018). From Rod-like to Disc-like Gay–Berne
Biaxial Nematics and Back. Liq. Cryst..

[6] Zannoni C. (2001). Computer Simulation
and Molecular Design of Model Liquid Crystals. J. Mater. Chem..

[7] Wilson M. R. (2007). Molecular
Simulation of Liquid Crystals: Progress towards a Better Understanding
of Bulk Structure and the Prediction of Material Properties. Chem. Soc. Rev..

[8] Allen M. P. (2019). Molecular
Simulation of Liquid Crystals. Mol. Phys..

[9] Vanakaras A. G., Photinos D. J. (2018). Atomistic Simulations
of Nematic Phases Formed by Cyano-Biphenyl
Dimers. Liq. Cryst..

[10] Takemoto K., Ishii Y., Washizu H., Kim K., Matubayasi N. (2022). Simulating
the Nematic-Isotropic Phase Transition of Liquid Crystal Model via
Generalized Replica-Exchange Method. J. Chem.
Phys..

[11] Palermo M. F., Pizzirusso A., Muccioli L., Zannoni C. (2013). An Atomistic Description
of the Nematic and Smectic Phases of 4-n-Octyl-4′ Cyanobiphenyl
(8CB). J. Chem. Phys..

[12] Tiberio G., Muccioli L., Berardi R., Zannoni C. (2009). Towards in Silico Liquid
Crystals. Realistic Transition Temperatures and Physical Properties
for n-Cyanobiphenyls via Molecular Dynamics Simulations. ChemPhysChem.

[13] Yu G., Wilson M. R. (2022). All-Atom Simulations
of Bent Liquid Crystal Dimers:
The Twist-Bend Nematic Phase and Insights into Conformational Chirality. Soft Matter.

[14] Greff
Da Silveira L., Livotto P. R., Padula D., Vilhena J. G., Prampolini G. (2022). Accurate Quantum-Mechanically Derived Force-Fields
through a Fragment-Based Approach: Balancing Specificity and Transferability
in the Prediction of Self-Assembly in Soft Matter. J. Chem. Theory Comput..

[15] Prampolini G., Greff Da Silveira L., Vilhena J. G., Livotto P. R. (2022). Predicting Spontaneous
Orientational Self-Assembly: In Silico Design of Materials with Quantum
Mechanically Derived Force Fields. J. Phys.
Chem. Lett..

[16] de
Mello M., Wilson M. R., Araki T. (2025). Impact of Charge Distribution
on the Stability of Ferroelectric Nematic Liquid Crystals. Soft Matter.

[17] Ogita S., Ishii Y., Watanabe G., Washizu H., Kim K., Matubayasi N. (2025). Atomistic
Analysis of Nematic Phase Transition in 4-Cyano-4′-n-Alkyl
Biphenyl Liquid Crystals: Sampling for the First-Order Phase Transition
and the Free-Energy Decomposition. J. Chem.
Phys..

[18] Hobbs J., Gibb C. J., Pociecha D., Szydłowska J., Górecka E., Mandle R. J. (2025). Polar Order in a Fluid Like Ferroelectric
with a Tilted Lamellar Structure – Observation of a Polar Smectic
C (SmCP) Phase. Angew. Chem., Int. Ed..

[19] Pizzirusso A., Di Pietro M. E., De Luca G., Celebre G., Longeri M., Muccioli L., Zannoni C. (2014). Order and Conformation
of Biphenyl
in Cyanobiphenyl Liquid Crystals: A Combined Atomistic Molecular Dynamics
and 1H NMR Study. ChemPhysChem.

[20] Weber A. C. J., Burnell E. E., Meerts W. L., De Lange C. A., Dong R. Y., Muccioli L., Pizzirusso A., Zannoni C. (2015). Molecular Dynamics
and 1H NMR of n -Hexane in Liquid Crystals. J. Chem. Phys..

[21] Vilhena J. G., Greff Da Silveira L., Livotto P. R., Cacelli I., Prampolini G. (2021). Automated
Parameterization of Quantum Mechanically Derived Force Fields for
Soft Materials and Complex Fluids: Development and Validation. J. Chem. Theory Comput..

[22] Maginn E. J., Messerly R. A., Carlson D. J., Roe D. R., Elliot J. R. (2020). Best Practices
for Computing Transport Properties 1. Self-Diffusivity and Viscosity
from Equilibrium Molecular Dynamics. Living
J. Comput. Mol. Sci..

[23] Wilson M. R., Yu G., Potter T. D., Walker M., Gray S. J., Li J., Boyd N. J. (2022). Molecular
Simulation Approaches to the Study of Thermotropic
and Lyotropic Liquid Crystals. Crystals.

[24] Grasselli F. (2022). Investigating
Finite-Size Effects in Molecular Dynamics Simulations of Ion Diffusion,
Heat Transport, and Thermal Motion in Superionic Materials. J. Chem. Phys..

[25] Celebi A. T., Jamali S. H., Bardow A., Vlugt T. J. H., Moultos O. A. (2021). Finite-Size
Effects of Diffusion Coefficients Computed from Molecular Dynamics:
A Review of What We Have Learned so Far. Mol.
Simul..

[26] de
Miguel E. (1993). System-Size Effects at the Isotropic-Nematic Transition from Computer
Simulation. Phys. Rev. E.

[27] Harris J. J., Pantelopulos G. A., Straub J. E. (2021). Finite-Size Effects
and Optimal System
Sizes in Simulations of Surfactant Micelle Self-Assembly. J. Phys. Chem. B.

[28] Paul T., Saha J. (2019). Computer Simulation Study of Novel Chiral Liquid Crystal Phases. Phys. Rev. Res..

[29] Castro-Román F., Benz R. W., White S. H., Tobias D. J. (2006). Investigation of
Finite System-Size Effects in Molecular Dynamics Simulations of Lipid
Bilayers. J. Phys. Chem. B.

[30] Skacej G., Zannoni C. (2021). The Nematic-Isotropic Transition of the Lebwohl–Lasher
Model Revisited. Philos. Trans. R. Soc..

[31] Lee J., Kosterlitz J. M. (1991). Finite-Size Scaling and Monte Carlo Simulations of
First-Order Phase Transitions. Phys. Rev. B.

[32] Vita F., Hegde M., Portale G., Bras W., Ferrero C., Samulski E. T., Francescangeli O., Dingemans T. (2016). Molecular
Ordering in the High-Temperature Nematic Phase of an All-Aromatic
Liquid Crystal. Soft Matter.

[33] Humpert A., Allen M. P. (2015). Elastic Constants and Dynamics in Nematic Liquid Crystals. Mol. Phys..

[34] Madsen L. A., Dingemans T. J., Poon C.-D., Samulski E. T. (2020). Exploring Ideality
and Reality in an Archetypal Rodlike Nematic Liquid Crystal. Liq. Cryst..

[35] Zafiropoulos N. A., Choi E. J., Dingemans T., Lin W., Samulski E. T. (2008). New All-Aromatic
Liquid Crystal Architectures. Chem. Mater..

[36] Olivier Y., Muccioli L., Zannoni C. (2014). Quinquephenyl The Simplest Rigid-Rod-like
Nematic Liquid Crystal, or Is It? An Atomistic Simulation. ChemPhysChem.

[37] Adenusi H., Muccioli L., Lanciotti M., Hegde M., Dingemans T. J., Samulski E. T., Vita F., Francescangeli O. (2025). Insights into
the Nanostructuring and Phase Behaviour of an All-Aromatic Prototypical
Nematic Liquid Crystal. Phys. Chem. Chem. Phys..

[38] Hindson J. C., Ulgut B., Friend R. H., Greenham N. C., Norder B., Kotlewski A., Dingemans T. J. (2010). All-Aromatic Liquid Crystal Triphenylamine-Based
Poly­(Azomethine)­s as Hole Transport Materials for Opto-Electronic
Applications. J. Mater. Chem..

[39] Rothera J. G., Yu J., AlNajm K., Butrus R., Ahangari-Bashash E., Watanabe L. K., Rawson J. M., Dmitrienko A., Vukotic V. N., Eichhorn S. H. (2025). Core-Only Calamitic Liquid Crystals:
Molecular Design and Optoelectronic Properties. Chem.—Asian J..

[40] Francescangeli O., Vita F., Samulski E. T. (2014). The Cybotactic Nematic Phase of Bent-Core
Mesogens: State of the Art and Future Developments. Soft Matter.

[41] Francescangeli O., Vita F., Ferrero C., Dingemans T., Samulski E. T. (2011). Cybotaxis Dominates the Nematic Phase
of Bent-Core
Mesogens: A Small-Angle Diffuse X-Ray Diffraction Study. Soft Matter.

[42] Sokolowska D., Moscicki J. K. (1996). Theory of Translational
Diffusion in the Nematic Phase. Phys. Rev. E.

[43] Kato T., Yoshio M., Ichikawa T., Soberats B., Ohno H., Funahashi M. (2017). Transport of Ions and Electrons in
Nanostructured Liquid
Crystals. Nat. Rev. Mater..

[44] Kondratenko K., Boussoualem Y., Longuemart S., Daoudi A. (2018). Ionic Transport in
Nematic Liquid Crystals and Alignment Layer Effects on Electrode Polarization. J. Chem. Phys..

[45] Dvinskikh S. V., Furó I., Zimmermann H., Maliniak A. (2002). Anisotropic Self-Diffusion
in Thermotropic Liquid Crystals Studied by 1H and 2H Pulse-Field-Gradient
Spin-Echo NMR. Phys. Rev. E.

[46] Dvinskikh S. V. (2020). Nuclear
Magnetic Resonance Studies of Translational Diffusion in Thermotropic
Ionic Liquid Crystals. Liq. Cryst..

[47] Kruger G. J. (1982). Diffusion
in Thermotropic Liquid Crystals. Phys. Rep..

[48] Yeh I. C., Hummer G. (2004). System-Size Dependence
of Diffusion Coefficients and
Viscosities from Molecular Dynamics Simulations with Periodic Boundary
Conditions. J. Phys. Chem. B.

[49] Phillips J. C., Hardy D. J., Maia J. D. C., Stone J. E., Ribeiro J. V., Bernardi R. C., Buch R., Fiorin G., Hénin J., Jiang W., McGreevy R., Melo M. C. R., Radak B. K., Skeel R. D., Singharoy A., Wang Y., Roux B., Aksimentiev A., Luthey-Schulten Z., Kalé L. V., Schulten K., Chipot C., Tajkhorshid E. (2020). Scalable Molecular
Dynamics on CPU and GPU Architectures with NAMD. J. Chem. Phys..

[50] Berendsen H. J. C., Postma J. P. M., Van
Gunsteren W. F., Dinola A., Haak J. R. (1984). Molecular
Dynamics with Coupling to an External Bath. J. Chem. Phys..

[51] Besler B. H., Merz K. M., Kollman P. A. (1990). Atomic
Charges Derived from Semiempirical
Methods. J. Comput. Chem..

[52] Frisch, M. J. ; Trucks, G. W. ; Schlegel, H. B. ; Scuseria, G. E. ; Robb, M. A. ; Cheeseman, J. R. ; Scalmani, G. ; Barone, V. ; Petersson, G. A. Gaussian 16, Revision C.01; Gaussian, Inc.: Wallingford, CT, 2016.

[53] Grossfield A., Zuckerman D. (2009). Chapter 2
Quantifying Uncertainty and Sampling Quality
in Biomolecular Simulations. Annu. Rep. Comput.
Chem..

[54] Flyvbjerg H., Petersen H. G. (1989). Error Estimates
on Averages of Correlated Data. J. Chem. Phys..

[55] Berardi R., Muccioli L., Zannoni C. (2004). Can Nematic Transitions Be Predicted
by Atomistic Simulations? A Computational Study of the Odd-Even Effect. ChemPhysChem.

[56] Boyd N. J., Wilson M. R. (2015). Optimization of
the GAFF Force Field to Describe Liquid
Crystal Molecules: The Path to a Dramatic Improvement in Transition
Temperature Predictions. Phys. Chem. Chem. Phys..

[57] Pizzirusso A., Savini M., Muccioli L., Zannoni C. (2011). An Atomistic Simulation
of the Liquid-Crystalline Phases of Sexithiophene. J. Mater. Chem..

[58] Palczynski K., Heimel G., Heyda J., Dzubiella J. (2014). Growth and
Characterization of Molecular Crystals of Para -Sexiphenyl by All-Atom
Computer Simulations. Cryst. Growth Des..

[59] Binder K., Landau D. P. (1984). Finite-Size Scaling At First-Order Phase Transitions. Phys. Rev. B.

[60] Franklin W. (1975). Theory of
Translational Diffusion in Nematic Liquid Crystals. Phys. Rev. A.

[61] Chu K.-S., Moroi D. S. (1975). Self-Diffusion in
Nematic Liquid Crystals. J. Phys. Arch..

[62] Chakrabarti D., Bagchi B. (2006). Anisotropic Translational Diffusion
in the Nematic
Phase: Dynamical Signature of the Coupling between Orientational and
Translational Order in the Energy Landscape. Phys. Rev. E..

[63] Cifelli M., Domenici V., Dvinskikh S. V., Veracini C., Zimmermann H. (2012). Translational
Self-Diffusion in the Smectic Phases of 4-n-Pentyl-4′-Cyanobiphenyl
as Studied by 2H NMR. Phase Transitions.

[64] Cifelli M., Cinacchi G., De Gaetani L. (2006). Smectic Order
Parameters from Diffusion
Data. J. Chem. Phys..

[65] Volino F., Dianoux A. J., Heidemann A. (1979). Self-Diffusion
Coefficients of TBBA:
Comparison between Neutron and NMR Results. J. Phys. Arch..

