# Supplementary material for: System-Size Effects on the Molecular Dynamics Simulation of an All-Aromatic Liquid Crystal
Source: ACS Phys Chem Au. 2026 May 18;6(4):773–82. doi: 10.1021/acsphyschemau.6c00022 (PMC13397453; doi:10.1021/acsphyschemau.6c00022)
Supplement: Supplementary file 1 [file pg6c00022_si_001.pdf]

Supporting Information

System-Size Effects on the Molecular Dynamics Simulation of an All-Aromatic Liquid Crystal

Henry Adenusi[1#*], Francesco Vita[1#], Luca Muccioli[2], Matteo Lanciotti[1], Oriano Francescangeli[1*]

[1]Department of Science and Engineering of Materials, Environment and Urban Planning, Marche Polytechnic University, Via Brecce Bianche, 60131, Ancona, Italy

[2]Department of Industrial Chemistry, University of Bologna, Via Gobetti 85, 40129, Bologna, Italy

[#]HA and FV contributed equally and share first authorship

*E-mail: o.francescangeli@staff.univpm.it, h.i.f.adenusi@staff.univpm.it

Supporting Information

S1. NAMD topology file (CHARMM format)

```
AUTOgenerate ANGLES DIHEDRAL

MASS 1  CA  12.0110  CA
MASS 2  CP  12.0110  CP
MASS 3  HA   1.0079  HA

RESIDUE PNP  0.0
# b3lyp/6-311++g(d,p) Pop=Espdipole charges, symmetrized

 group
 atom  C1      CA   -0.160075
 atom  H2      HA   +0.130800
 atom  C3      CA   -0.126375
 atom  H4      HA   +0.128200
 atom  C5      CA   -0.139550
 atom  H6      HA   +0.131100
 atom  C7      CA   -0.126375
 atom  H8      HA   +0.128200
 atom  C9      CA   -0.160075
 atom  H10     HA   +0.130800
 atom  C11     CP   +0.050100
 atom  C12     CP   +0.084400
 atom  C13     CA   -0.199800
 atom  C14     CA   -0.094600
 atom  H15     HA   +0.121100
 atom  H16     HA   +0.135550
 atom  C17     CA   -0.199800
 atom  H18     HA   +0.135550
 atom  C19     CA   -0.094600
 atom  H20     HA   +0.121100
 atom  C21     CP   -0.015050
 atom  C22     CP   +0.073900
 atom  C23     CA   -0.312050
 atom  H24     HA   +0.166000
 atom  C25     CA   -0.140650
 atom  H26     HA   +0.138800
 atom  C27     CA   -0.329000
 atom  H28     HA   +0.180100
 atom  C29     CA   +0.242300
 atom  C30     CA   -0.312050
 atom  H31     HA   +0.166000
 atom  C32     CA   +0.242300
 atom  C33     CA   -0.329000
 atom  H34     HA   +0.180100
 atom  C35     CA   -0.140650
 atom  H36     HA   +0.138800
 atom  C37     CP   +0.073900
 atom  C38     CP   -0.015050
 atom  C39     CA   -0.094600
 atom  C40     CA   -0.199800
 atom  H41     HA   +0.135550
```

```
atom  H42     HA   +0.121100
atom  C43     CA   -0.094600
atom  H44     HA   +0.121100
atom  C45     CA   -0.199800
atom  H46     HA   +0.135550
atom  C47     CP   +0.084400
atom  C48     CP   +0.050100
atom  C49     CA   -0.160075
atom  H50     HA   +0.130800
atom  C51     CA   -0.126375
atom  H52     HA   +0.128200
atom  C53     CA   -0.139550
atom  H54     HA   +0.131100
atom  C55     CA   -0.126375
atom  H56     HA   +0.128200
atom  C57     CA   -0.160075
atom  H58     HA   +0.130800

bond C1     H2
bond C1     C3
bond C1     C11
bond C3     H4
bond C3     C5
bond C5     H6
bond C5     C7
bond C7     H8
bond C7     C9
bond C9     H10
bond C9     C11
bond C11    C12
bond C12    C13
bond C12    C17
bond C13    C14
bond C13    H16
bond C14    H15
bond C14    C21
bond C17    H18
bond C17    C19
bond C19    H20
bond C19    C21
bond C21    C22
bond C22    C23
bond C22    C25
bond C23    H24
bond C23    C32
bond C25    H26
bond C25    C27
bond C27    H28
bond C27    C29
bond C29    C30
bond C29    C32
bond C30    H31
bond C30    C37
```

```
 bond C32    C33
 bond C33    H34
 bond C33    C35
 bond C35    H36
 bond C35    C37
 bond C37    C38
 bond C38    C39
 bond C38    C43
 bond C39    C40
 bond C39    H42
 bond C40    H41
 bond C40    C47
 bond C43    H44
 bond C43    C45
 bond C45    H46
 bond C45    C47
 bond C47    C48
 bond C48    C49
 bond C48    C57
 bond C49    H50
 bond C49    C51
 bond C51    H52
 bond C51    C53
 bond C53    H54
 bond C53    C55
 bond C55    H56
 bond C55    C57
 bond C57    H58
```

S2. NAMD input file

```
#--- integrator
numsteps                15000000
timestep                1
nonbondedFreq           1
fullElectFrequency      2
stepspercycle           20

#--- Parameter options
paratypeCharmm    on
parameters        gaff2_charmm.prm
structure         ppnpp_12600mol.psf
exclude           scaled1-4
1-4scaling        0.83333
cutoff            12.
switching         on
switchdist        11.5
pairlistdist      15.
```

Supporting Information

```
#--- Thermodynamic
BerendsenPressure                   on
BerendsenPressureTarget             1.01325
BerendsenPressureCompressibility    0.0000457
BerendsenPressureRelaxationTime     20000.
BerendsenPressureFreq               1000
useFlexibleCell                     yes
useGroupPressure                    yes
rescaleTemp       700. K
rescalefreq       20
COMmotion         no
#langevin         on
#langevinTemp     1. K
#langevinDamping  0.2

#--- PBC
cellBasisVector1  141.254     0.          0.
cellBasisVector2  0.          485.516     0.
cellBasisVector3  0.          0.          142.105
extendedSystem    ppnppT690_restart.xsc
wrapAll           no

#--- PME
dielectric        1
PME               on
PMEGridSpacing    1.5
ZeroMomentum      yes

#--- Input coords
#temperature      700. K
coordinates       PPNPP_12600mol.pdb
bincoordinates    ppnppT690_restart.coor
binvelocities     ppnppT690_restart.vel

#--- Output & Restart
binaryoutput      no
outputname        ppnppT700
binaryrestart     yes
restartname       ppnppT700_restart
restartfreq       10000
DCDfile           ppnppT700.dcd
DCDfreq           20000
XSTfreq           20000

#---  Standard Output
outputEnergies    1000
```

Supporting Information

S3. Equilibration analysis

To demonstrate the equilibration of our samples, we provide a few representative examples of computed quantities (density, orientational and positional order parameter) versus time for the largest system at T = 690 K, close to the SmA-N phase transition, and at T = 750 K, close to the N-I phase transition. The starting state for the simulation at T = 690 K was prepared by spatially replicating the equilibrated sample for N = 350 molecules at the same temperature as described in the Methods section of the manuscript. The simulation at T = 750 K used as starting state the equilibrated sample for N = 12600 at T = 745 K.

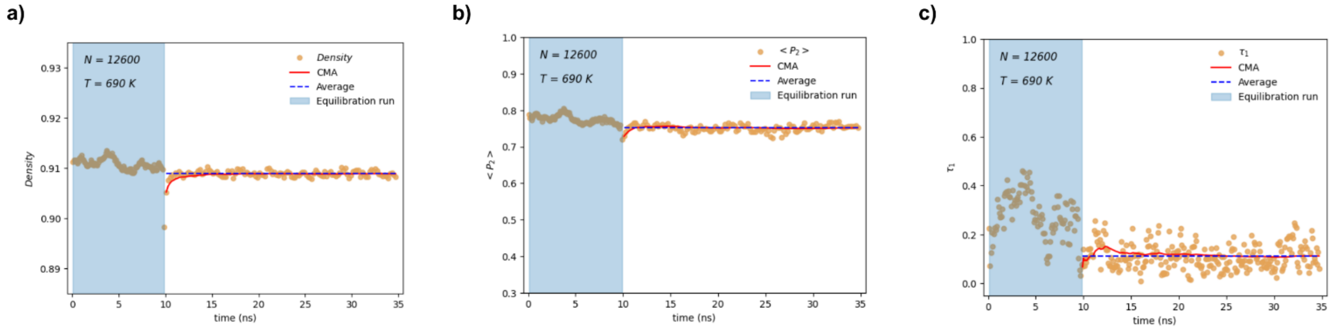

Figure S1(a-c). Plots of computed quantities versus time during the equilibration run (region highlighted in blue) and the production run at T = 690 K for N = 12600. The dashed blue line and the red solid line indicate the average value and the cumulative moving average (CMA), respectively, computed over the production run: (a) density; (b) orientational order parameter; (c) positional order parameter.

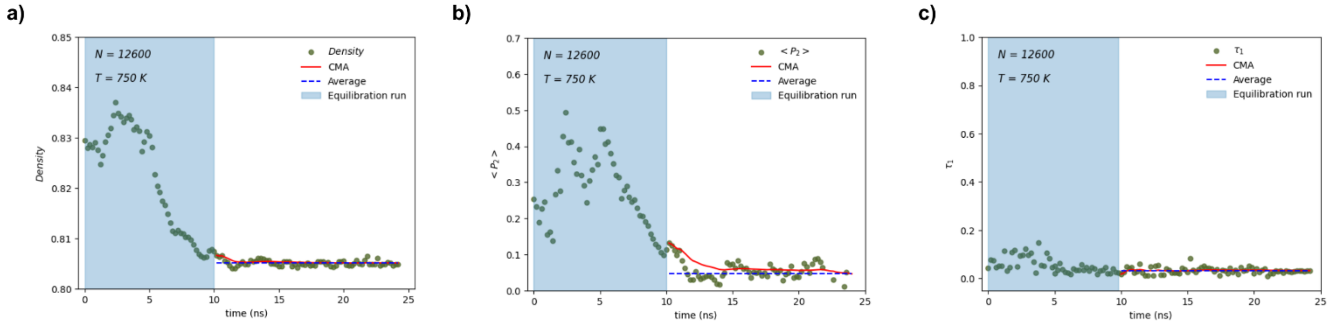

Figure S2(a-c). Plots of computed quantities vs time during the equilibration run (region highlighted in blue) and the production run at T = 750 K for N = 12600. The dashed blue line and the red solid line indicate the average value and the cumulative moving average (CMA), respectively, computed over the production run: (a) density; (b) orientational order parameter; (c) positional order parameter.

S4. Computation of translational diffusion coefficients

For each direction in the director frame, the translational self-diffusion coefficient D was evaluated by least-square fitting with a straight line the corresponding mean square displacement versus time profile, from $t_i = 0$ to $t_f = 1$ ns. Assuming that the trajectories are saved every time interval Δt (here, 20 ps), the displacement for molecule i between couples of individual configurations j, k separated by time $(k-j)\Delta t$ were calculated in the phase director frame through the average rotation matrix R_{jk} from the box to the director frame, defined by computing the Euler angles from the rotation matrices of frames $j,\ k$ and computing the new rotation matrix from the average of those angles. In the case of constant pressure (NpT) simulations, also the average box side vector $\mathbf{L}_{jk} = \frac{\mathbf{L}_k + \mathbf{L}_j}{2}$ enters the calculation, following Schmitz et al. (https://pubs.acs.org/doi/10.1021/jp990761s):

$$\Delta \mathbf{r}_i[(k-j)\Delta t] = R_{jk}\mathbf{L}_{jk} \odot \left(\mathbf{r}_{i,k} \oslash \mathbf{L}_k - \mathbf{r}_{i,j} \oslash \mathbf{L}_j\right).$$

Supporting Information

The mean squared displacement vector $\langle \Delta \mathbf{r}^2 \rangle$ at any specific time was obtained as an average over all molecules and time origins of the element-wise square of $\Delta \mathbf{r}_i$:

$$\langle \Delta \mathbf{r}^2 (m\Delta t) \rangle = \frac{1}{N} \frac{1}{(M-m)} \sum_{j=1}^{M-m} \sum_{k=j+m}^{M} \sum_{i}^{N} \Delta \mathbf{r}_i[(k-j)\Delta t] \odot \Delta \mathbf{r}_i[(k-j)\Delta t],$$

where $m = k - j$, N is the number of molecules and M the number of configurations in the trajectory, and the standard symbols $\odot$, $\oslash$ indicate the element-wise product and division, respectively. The procedure was repeated for $m = 1, 2, \ldots, M-1$ to obtain a table of $\langle \Delta \mathbf{r}^2(t) \rangle$ at discrete time intervals with step Δt.

Then, following Einstein's equation, the isotropic diffusion coefficient D_{iso} was obtained by fitting with a straight line, in the time interval $[t_i, t_f]$, the sum of the three components of $\langle \Delta \mathbf{r}^2(t) \rangle$, and dividing the slope by 6. A similar procedure was applied separately to the three components x, y, z of $\langle \Delta \mathbf{r}^2(t) \rangle$, finally getting $D_{||}$ as half the slope of the line fitting the z component, and $D_{\perp}$ from half the average slope of the lines fitting separately the x and y components.

The procedure gives reliable results for the single components of $\langle \Delta \mathbf{r}^2(t) \rangle$ only if the time scale of the rotation of the director frame is longer than $t_f - t_i$, and then $\mathrm{R}_k \approx \mathrm{R}_j$. In addition, the number of available time intervals and, accordingly, the reliability of the values decrease with m, therefore one should choose the interval $t_f - t_i$ short enough to achieve good statistics and limited rotation of the phase director, but long enough to reach the diffusive behavior. The exact choice is somehow arbitrary: for our systems we chose $t_i = 0$ and $t_f = 1$ ns, but slightly different diffusion coefficient values can be obtained with other choices of t_f, as shown in Figure S3.

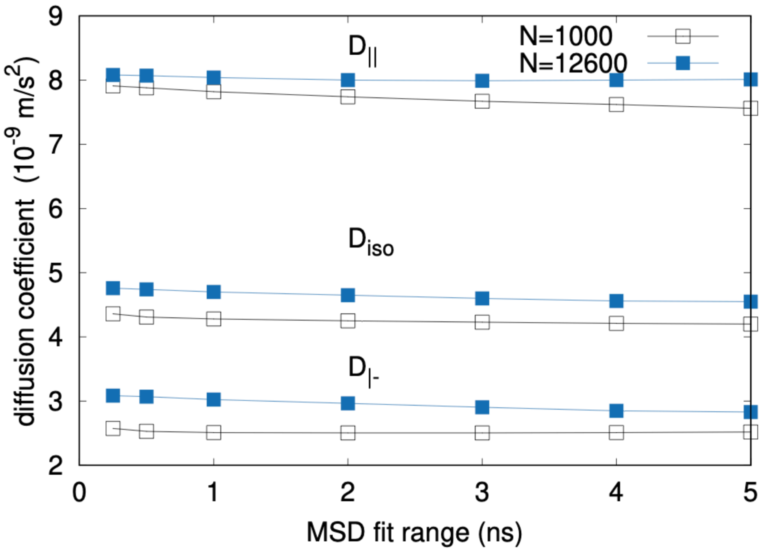

Figure S3. Diffusion coefficients obtained at 730 K for different system sizes (white squares N = 1000, blue squares N = 12600) and different choices of the time range $[0, t_f]$ for fitting the mean square displacement.

To estimate the error in the self-diffusion coefficient values, we applied the block average method by splitting the trajectories at 730 K in blocks of increasing size b (see e.g. the resulting MDS in Figure S4 for $b = 100$), following Chitra and Yashonat (https://doi.org/10.1021/jp9703059). For each block size we calculated the average of the diffusion coefficients and its standard deviation and calculated, as an estimator of the standard error of the mean, the standard deviation of the coefficients divided by the square root of the number of blocks, $\sqrt{N_b}$. Although specific and much longer runs would be required to achieve the full convergence of the block average method, the error values (Table S1) hint at a standard error of the order of 1%. Therefore, we conclude that the differences in D_{iso}, $D_{\perp}$, $D_{||}$ that we register from one sample size and another (c.f. Table S2) are statistically meaningful and a true effect of the different number of molecules N.

To this regard, one could also argue that the sample size effects in the D_{iso}, $D_{\perp}$, $D_{||}$ values are mainly due to the density differences between the samples and to the volume fluctuations that follow the choice of adopting the *NpT* ensemble, *i.e.* that different sample sizes, if studied at the same density, would yield the same values of the diffusion coefficient. To investigate this effect, we conducted constant volume (*NVT*) simulations for the three system sizes at *T* = 730 K (same length of the corresponding *NpT* simulations). This temperature, where the nematic order parameter calculated from *NpT* simulations is rather similar for the three sample sizes, was selected to allow a fairer comparison of $D_{\perp}$ and $D_{||}$, since their values and ratio are strongly dependent on $\langle P_2 \rangle$.

The results obtained from the *NVT* simulations are reported in Table S2, alongside the corresponding ones in the *NpT* ensemble. It can be noticed that the different ensembles give rise to non-negligible variations of the diffusion coefficients and that, even for *NpT* simulations, the different samples sizes lead to different results, with the largest sample always showing faster diffusion and larger diffusion anisotropy, despite having also the lowest nematic order parameter.

Table S1. Average values and standard errors for diffusion coefficients (units: 10^{-9} m/s^2) as calculated by splitting the original trajectories in $\boldsymbol{N_b}$ blocks of increasing block size $\boldsymbol{b}$ (units: number of frames, saved every $\Delta \boldsymbol{t} = \mathbf{20}$ ps). The trajectories are composed of 1700, 1000, and 850 frames for $\boldsymbol{N}$ = 350, 1000, 12600, respectively.

**N**	N_b	**b**	D_{iso}	$\sigma_{iso}/\sqrt{N_b}$	$D_{\perp}$	$\sigma_{\perp}/\sqrt{N_b}$	$D_{\parallel}$	$\sigma_{\parallel}/\sqrt{N_b}$
350	17	100	4.26	0.042	2.47	0.041	7.87	0.097
	8	213	4.26	0.036	2.55	0.078	7.67	0.120
	4	425	4.26	0.015	2.54	0.057	7.71	0.091
	2	850	4.27	0.009	2.45	0.021	7.91	0.069
1000	10	100	4.26	0.034	2.50	0.022	7.77	0.082
	5	200	4.28	0.019	2.51	0.027	7.83	0.004
	4	250	4.27	0.028	2.50	0.023	7.81	0.051
	2	500	4.28	0.034	2.51	0.027	7.82	0.048
12600	9	100	4.71	0.041	3.05	0.065	8.05	0.043
	4	213	4.71	0.039	3.04	0.060	8.05	0.035
	2	425	4.69	0.008	3.01	0.021	8.05	0.036

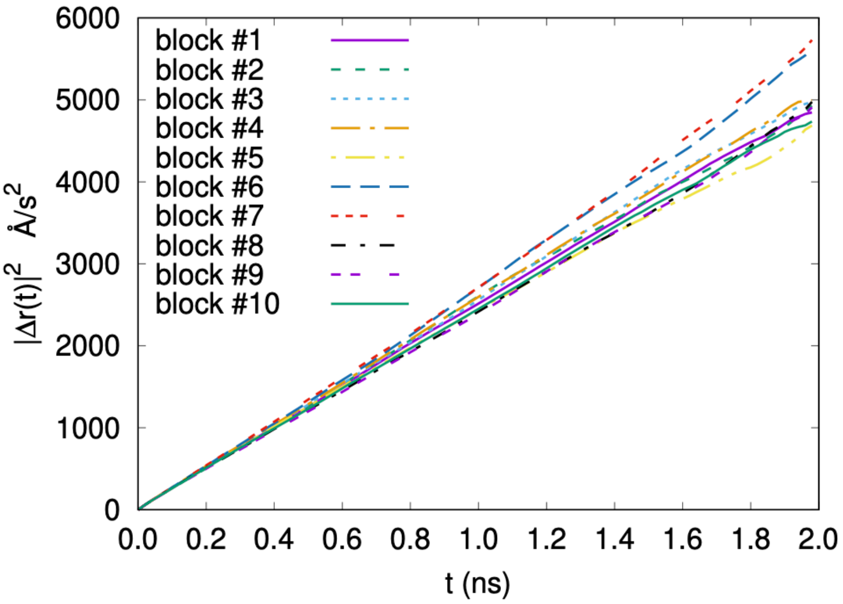

Figure S4. Mean square displacements versus time at 730 K evaluated for 10 different trajectory blocks, each 2-ns long, for the $N = 350$ system. The figure suggests that a block size of 2 ns (i.e., $b = 100$ frames per block) is too short for evaluating the coefficients, since there is a certain variability in the slopes of the MDS curves of the different blocks.

Table S2. Comparison of the average values for diffusion coefficients (units: 10^{-9} m/s^2) calculated at 730 K in the *NpT* and *NVT* ensembles. The corresponding density (g/cm^3), nematic and smectic order parameter are also reported.

**N**	**ensemble**	D_{iso}	$D_{\perp}$	$D_{\|\|}$	$D_{\|\|}/D_{\perp}$	**density**	$\langle P_2 \rangle$	τ_1
350	*NpT*	4.30	2.90	7.10	2.45	0.859	0.65	0.13
1000	*NpT*	4.20	2.69	7.22	2.68	0.857	0.62	0.08
12600	*NpT*	4.55	2.82	8.01	2.84	0.856	0.58	0.04
350	*NVT*	4.10	2.57	7.17	2.79	0.855	0.66	0.13
1000	*NVT*	4.36	2.78	7.53	2.71	0.855	0.61	0.11
12600	*NVT*	4.56	2.85	7.98	2.80	0.855	0.57	0.05

S5. Determination of transition temperatures

Phase transitions were determined from the analysis of the temperature dependence of the relevant order parameters, <P_2> and τ_1 for the N-I and SmA-N phase transition, respectively. The computed values of the order parameters as a function of temperature were fitted with a model curve $y(T)$ obtained by multiplying a sigmoid centered in T_0 by a straight line of slope b:

$$y(T) = \left\{A_0 + \Delta A \frac{1}{1+\exp[k(T-T_0)]}\right\}[1 + b(T - T_0)],$$

with A_0, ΔA, k, b and T_0 being the fit parameters. This choice provides a faithful description of the trend of both order parameters across the transitions. The N-I transition temperature was identified with the value of T_0 provided by the fit, as shown in Figure S3(a-c): for small values of the parameter b (as it is in our case), this essentially coincides with the inflection point of the curve $y(T)$.

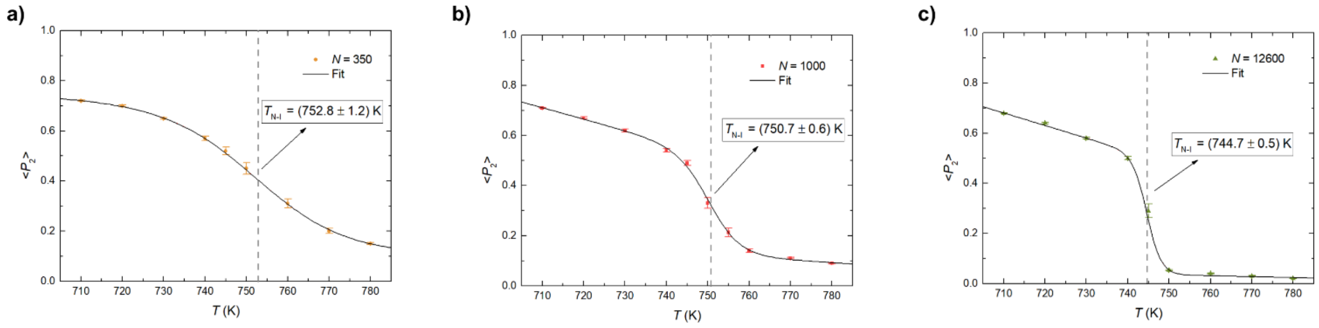

Figure S5(a-c). Fit of the orientational order parameter <P_2> across the N-I phase transition: (a) N = 350; (b) N = 1000; (c) N = 12600. A vertical dashed line indicates the transition temperature. Error bars indicate SEs (where not visible, they are smaller than data symbols).

Contrary to the weakly first order N-I phase transition, the SmA-N phase transition is found to be second order, in agreement with the theory. Accordingly, due to the absence an abrupt change of the positional order parameter τ_1, we chose to identify the SmA-N transition temperature with the point $T_{SmA-N} = T_0 + \frac{1}{k}\log\frac{0.9}{0.1}$ where the sigmoid drops by 90% of its maximum, as shown in Figure S4(a-c), rather than with the center of the sigmoid as done for the N-I phase transition.

Supporting Information

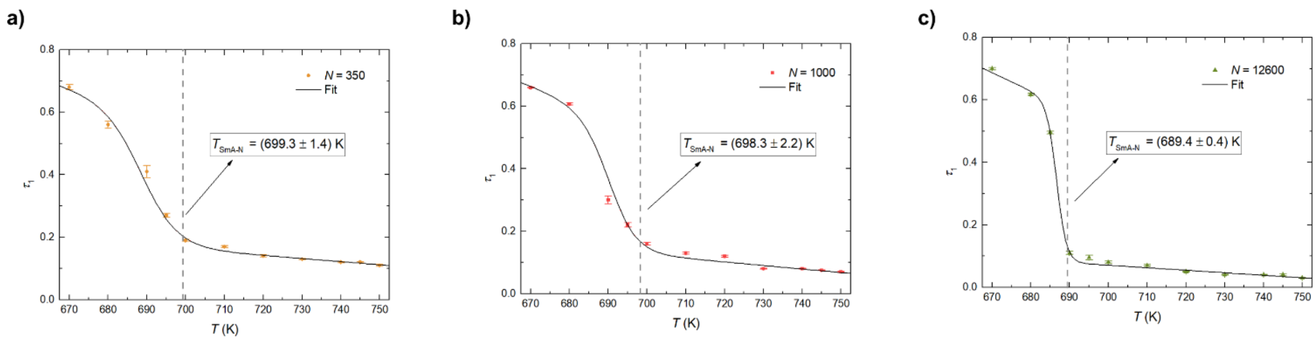

Figure S6(a-c). Fit of the positional order parameter τ_1 across the SmA-N phase transition: (a) *N* = 350; (b) *N* = 1000; (c) *N* = 12600. A vertical dashed line indicates the transition temperature. Error bars indicate SEs (where not visible, they are smaller than data symbols).

S6. Determination of transition enthalpies

Transition enthalpies Δ*H* were calculated as follows. First, for each phase, we performed a separate linear fit of the enthalpy data *H*(*T*) as a function of temperature. We then used these linear trends to extrapolate the *H* values of each phase at the transition temperature and calculated Δ*H* as their difference. For instance, Δ*H* at the N-I phase transition was calculated as $\Delta H = (q_I + m_I T_{NI}) - (q_N + m_N T_{NI})$, where m_I, q_I and m_N, q_N are the fit coefficients (slope and intercept) obtained from fitting *H*(*T*) in the I and N phase, respectively, and T_{NI} is the transition temperature determined as described in the previous section.

The uncertainty on Δ*H* was calculated through error propagation, also considering the covariance between the *q* and *m* parameters of each linear fit and the uncertainty on the transition temperature. For the N-I phase transition, the uncertainty was thus calculated as:

$$\sigma_{\Delta H} = \sqrt{\sigma_{q_I}^2 + \sigma_{q_N}^2 + T_{NI}^2\left(\sigma_{m_I}^2 + \sigma_{m_N}^2\right) + 2T_{NI}[\mathrm{Cov}(q_I, m_I) + \mathrm{Cov}(q_N, m_N)] + (m_I - m_N)^2\sigma_{T_{NI}}^2}.$$

S7. Molecular aspect ratio

Table S3. Values of simulated aspect ratio *Q* (*L*/*d*) as a function of temperature.

***T* / K**	*N* = 350	*N* = 1000	*N* = 12600
670	3.72	3.70	3.70
680	3.69	3.67	3.67
690	3.67	3.64	3.64
695	3.66	3.63	3.63
700	3.64	3.63	3.62
710	3.63	3.61	3.61
720	3.62	3.60	3.60
730	3.61	3.59	3.59
740	3.60	3.58	3.58
745	3.59	3.57	3.57
750	3.58	3.56	3.56
760	3.57	3.55	3.55
770	3.56	3.54	3.54
780	3.55	3.53	3.53

S8. Arrhenius fit of diffusion coefficients

The Arrhenius law $D = D_0 e^{-E_a/RT}$ has been used to fit the diffusion coefficients D_{iso}, $D_{\parallel}$ and $D_{\perp}$ as described in the main text. The values of the activation energy E_a so obtained are reported in Table 2. Here we report the values of the D_0 parameters.

Table S4. Values of parameter D_0 obtained from fitting the diffusion data in the nematic-isotropic (N-I) and smectic A (SmA) regions.

D_0 (m^2/s)	**N = 350**			**N = 1000**			**N = 12600**		
	D_{iso}	$D_{\parallel}$	$D_{\perp}$	D_{iso}	$D_{\parallel}$	$D_{\perp}$	D_{iso}	$D_{\parallel}$	$D_{\perp}$
**N-I**	$(1.50 \pm 0.16)\ 10^{-6}$	$(10 \pm 5)\ 10^{-7}$	$(3.49 \pm 0.06)\ 10^{-5}$	$(1.7 \pm 0.3)\ 10^{-6}$	$(1.0 \pm 1.4)\ 10^{-6}$	$(1.8 \pm 0.7)\ 10^{-5}$	$(3.1 \pm 0.5)\ 10^{-6}$	$(1.1 \pm 0.8)\ 10^{-6}$	$(4.0 \pm 1.0)\ 10^{-5}$
**SmA**	$(6 \pm 7)\ 10^{3}$	$(1 \pm 4)\ 10^{7}$	$(5 \pm 6)\ 10^{-2}$	$(1 \pm 3)\ 10^{7}$	$(0.4 \pm 2.9)\ 10^{14}$	$(3 \pm 3)\ 10^{-1}$	$(1 \pm 9)\ 10^{8}$	$(0.2 \pm 1.9)\ 10^{17}$	$(0.4 \pm 1.9)\ 10^{1}$
